# Prediction of late recurrence after curative-intent resection using MRI-measured spleen volume in patients with hepatocellular carcinoma and cirrhosis

**DOI:** 10.1186/s13244-024-01609-8

**Published:** 2024-02-02

**Authors:** Chongtu Yang, Jia Tan, Yidi Chen, Yanshu Wang, Yali Qu, Jie Chen, Hanyu Jiang, Bin Song

**Affiliations:** 1 Department of Radiology, West China Hospital, Sichuan University, Chengdu, 610041 China; 2 Department of Radiology, Sanya People’s Hospital, Sanya, Hainan China

**Keywords:** Hepatocellular carcinoma, Resection, Late recurrence, Spleen volume, Prognosis

## Abstract

**Background:**

Late recurrence of hepatocellular carcinoma (HCC) after liver resection is regarded as a de novo tumor primarily related to the severity of underlying liver disease. We aimed to investigate risk factors, especially spleen volume, associated with late recurrence in patients with HCC and cirrhosis.

**Methods:**

We retrospectively analyzed 301 patients with HCC and cirrhosis who received curative resection and preoperative MRI. Patients were followed for late recurrence for at least 2 years. Spleen volume was automatically measured on MRI with artificial intelligence techniques, and qualitative MRI imaging features reflecting tumor aggressiveness were evaluated. Uni- and multivariable Cox regression analyses were performed to identify independent predictors and a risk score was developed to predict late recurrence.

**Results:**

Eighty-four (27.9%) patients developed late recurrence during follow-up. Preoperative spleen volume was independently associated with late recurrence, and patients with a volume > 370 cm^3^ had significantly higher recurrence risk (hazard ratio 2.02, 95%CI 1.31–3.12, *p* = 0.002). Meanwhile, no qualitative imaging features were associated with late recurrence. A risk score was developed based on the APRI score, spleen volume, and tumor number, which had time-dependent area under the curve ranging from 0.700 to 0.751. The risk score at a cutoff of 0.42 allowed for the identification of two risk categories with distinct risk of late recurrence.

**Conclusions:**

Preoperative spleen volume on MRI was independently associated with late recurrence after curative-intent resection in patients with HCC and cirrhosis. A risk score was proposed for individualized risk prediction and tailoring of postoperative surveillance strategies.

**Critical relevance statement:**

Spleen volume measured on MRI with the aid of AI techniques was independently predictive of late HCC recurrence after liver resection. A risk score based on spleen volume, APRI score, and tumor number was developed for accurate prediction of late recurrence.

**Key points:**

• Preoperative spleen volume measured on MRI was independently associated with late recurrence after curative-intent resection in patients with HCC and cirrhosis.

• Qualitative MRI features reflecting tumor aggressiveness were not associated with late recurrence.

• A risk score based on spleen volume was developed for accurate prediction of late recurrence and risk stratification.

**Graphical Abstract:**

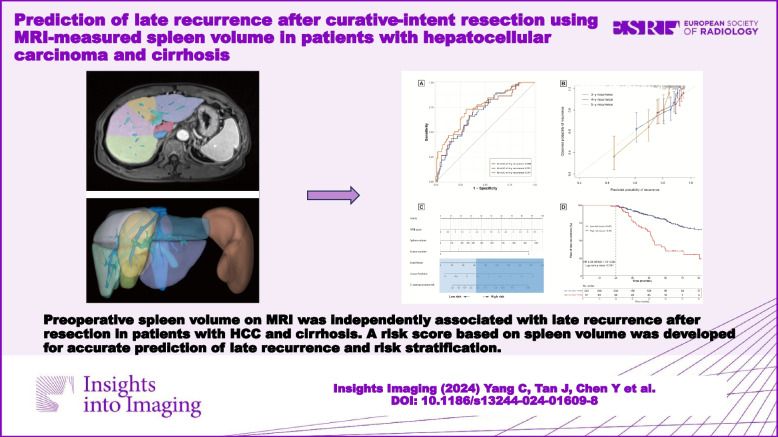

**Supplementary Information:**

The online version contains supplementary material available at 10.1186/s13244-024-01609-8.

## Introduction

Hepatocellular carcin oma (HCC) is the most common form of primary liver cancer, with over 80% of patients developing cirrhosis [1–4]. Surgical liver resection remains the mainstay of curative therapy for those with very early/early-stage tumor (Barcelona Clinic Liver Cancer [BCLC] 0/A) and preserved liver function [5, 6]. However, recurrence after resection adversely affects long-term prognosis, with a prevalence that has been reported to be as high as 60–70% within 5 years [7, 8].

Two types of HCC recurrence have been proposed: early recurrence (within 2 years after resection) and late recurrence (more than 2 years). Specifically, early recurrence has been associated with the aggressiveness of the primary HCC and is likely due to its intrahepatic metastasis. Conversely, late recurrence is more frequently regarded as the de novo multicentric tumor, which may be more related to the severity of the underlying liver disease [9, 10]. Identifying patients at increased risk of late recurrence can help tailor personalized treatment and follow-up strategy and consequently improve survival. To that end, cirrhosis has been reported as the predominant risk factor for late recurrence [9, 11], and a recent study found that spleen stiffness measured by transient elastography, which reflects the degree of portal hypertension, was the only predictor of late ecurrence [12]. However, spleen stiffness is not routinely measured during the preoperative workups of HCC. Furthermore, the added value of spleen measurement in patients with established cirrhosis remains unclear.

Similar to spleen stiffness, an increase in spleen volume has been positively associated with the severity of chronic liver disease (CLD) [13, 14] and is thus a potential predictive marker of late recurrence. Besides, the measurement of spleen volume is simpler than spleen stiffness with the aid of artificial intelligence (AI) techniques. On the other hand, in recent years contrast-enhanced MRI has shown promising prognostic utilities in HCC as it permits evaluation of the morphology, hemodynamics, metabolism, and function of the liver and tumor via multiparametric imaging sequences [15]. Therefore, MRI allows for accurate measurement of volumetric indices, simultaneously providing critical information regarding tumor aggressiveness.

Therefore, we aimed to explore risk factors of late HCC recurrence, especially spleen volume measured with AI techniques on preoperative MRI, and to develop a risk score for prediction of late recurrence and individualized risk stratification.

## Materials and methods

### Study population and data acquisition

The protocol of this retrospective study conforms to the ethical guidelines of the 1975 Declaration of Helsinki and was approved by the institutional review board. The study adhered to the TRIPOD guideline for developing and validating a prognostic model [16].

From January 2011 to May 2020, consecutive patients with HCC who underwent curative-intent resection and preoperative MRI scan at a tertiary academic hospital were screened. Inclusion criteria were as follows: (a) successfully followed up for at least 2 years (i.e., did not die, experience early recurrence, or lost to follow-up within 2 years), (b) with pathologically confirmed HCC, (c) with established cirrhosis determined based on liver biopsy or clinical history and typical imaging features. Exclusion criteria included the following: (a) received any prior procedure for the liver and/or spleen (e.g., transjugular intrahepatic portosystemic shunt, splenectomy, or partial splenic embolization); (b) presence of portal invasion and/or extrahepatic spread; (c) had any co-malignancies other than HCC; (d) had ruptured tumors; (e) received any adjuvant treatments before recurrence; (f) the interval between MRI and resection exceeded 1 month; and (g) had inadequate MRI image quality (e.g., severe artifact).

Baseline data were extracted from the electronic medical records, including patient demographics, clinical characteristics, laboratory results, and surgical information. For laboratory results, we collected the most recent values recorded before surgery. The pathological parameters included tumor number, maximum size, tumor differentiation, surgical margin width, microvascular invasion, and histological grade of liver fibrosis evaluated using either the Ishak or the Scheuer grading system [17, 18].

### MRI examination and qualitative imaging analysis

All MRI examinations were performed using either four 3.0-T systems or a 1.5-T system. Details of MRI acquisition protocols are provided in Supplementary materials.

Two abdominal radiologists (with 7 and 10 years of experience in liver MRI) who were blinded to all clinical, pathological, and follow-up data independently performed the imaging analyses. The following items were evaluated for each patient: (a) location, size, and number of tumors; (b) Liver Imaging Reporting and Data System (LI-RADS v.2018) major and ancillary features (except for those related to growth and ultrasound visibility); (c) other imaging features that reflect tumor aggressiveness (e.g., margin, growth subtypes, internal artery, bilobar involvement). Discrepancies were resolved by conducting a consensus review session with a third senior radiologist who had over 20 years of experience in liver MRI.

### Measurement of liver and spleen volumetric indices

All MRI images were transferred to a post-processing workstation dedicated to 3D volumetric analyses. An independent radiologist who was not involved in the imaging analyses performed volumetric analysis with a commercially available, fully automated volumetric software (SenseCare, Shanghai, China). Specifically, portal venous phase images were automatically segmented and the liver (including each lobe) and spleen volumes (cm^3^) were calculated by summing the corresponding consecutive areas and multiplying by the slice thickness (Supplementary Fig. 1). The calculation process took approximately 3 min for each case. The radiologist reviewed all segmentation generated by the software and corrected segmentation errors manually. The following volumetric indices were recorded: total liver volume, left/right/caudate lobe volume, spleen volume, and tumor volume.

### Follow-up and endpoint definition

As per protocol, a follow-up procedure was scheduled for all patients after surgery at 1 month, every 3 months for the first 2 years, and then every 6 months thereafter, or as clinically required, supplemented with telephone interviews every 6 months. Patients were followed until death, lost to follow-up, or the end of this study (May 1, 2022), and data were censored at the end of follow-up.

The primary endpoint for the study was late HCC recurrence, defined as radiological or pathological identification of local disease or distant metastasis at least 24 months after surgery. Secondary endpoints during follow-up included recurrence-free survival (RFS) and overall survival (OS).

### Statistical analysis

Quantitative variables were reported as means and standard deviation (SD) or median and interquartile range (IQR). Categorical variables were reported as frequencies and percentages. Comparisons between groups were conducted by *t*-test, Mann-Whiney test, chi-squared test, or Fisher’s exact test as appropriate. Correlation between volumetric indices, laboratory, and pathologic parameters was assessed with either Pearson or Spearman correlation. Kaplan–Meier curves with log-rank tests were used for survival analysis, and Cox proportional hazards model was used to estimate the multivariable-adjusted hazard ratios (HRs) and 95% confidence intervals (95%CIs). The non-linear association between spleen volume and risk of late recurrence was assessed using restricted cubic splines fitted in Cox regression model.

For the prediction of late recurrence, a risk prediction score was developed. Variables with a *p* value < 0.1 in the univariable Cox analysis were entered into the multivariable analysis. Single components of the established scores (e.g., Child–Pugh, MELD, FIB-4) were not entered simultaneously to avoid multicollinearity. A backward stepwise procedure based on the Akaike information criteria was used to simplify the model and identify the best subset of independent predictors. The final score was built by fitting a Cox model, with 5-year recurrence as the endpoint, and the coefficients estimated for each predictor were used as relative weights to compute the linear predictor.

We assessed the predictive accuracy of the score with discrimination and calibration. Discrimination was assessed with the time-dependent area under the receiver operator characteristic (td-AUC) curves. Calibration was assessed statistically by computing the Brier score and graphically by generating the calibration plot. Besides, the developed score was internally validated using bootstrap resampling method (with 1000 replicates) to examine optimism in score performance. To identify two risk categories with distinct risk of late recurrence, a cutoff value was used as the linear predictor of the final score at its 80^th^ percentile. Furthermore, the final score was applied for the prediction of different patterns of late recurrence (definitions are provided in the Supplementary materials).

Two sets of sensitivity analyses were implemented to examine the robustness of our results. First, Fine and Gray’s subdistribution hazards regression model was applied to evaluate the possible influence of competing events on the endpoint, taking death as a competing event for late recurrence. Second, to evaluate whether the discriminative ability of the developed score could be improved by parameters obtained after surgery (e.g., surgery-related and pathological indices), we included additional variables significantly associated with the endpoint (clinically or statistically) to the score individually.

Missing values were assumed to be missing at random and thus were imputed with multiple imputation with chained equations. A two-tailed *p* value < 0.05 was considered statistically significant. All statistical analyses were performed with R software (version 4.2.2).

## Results

### Baseline characteristics

**Fig. 1:**
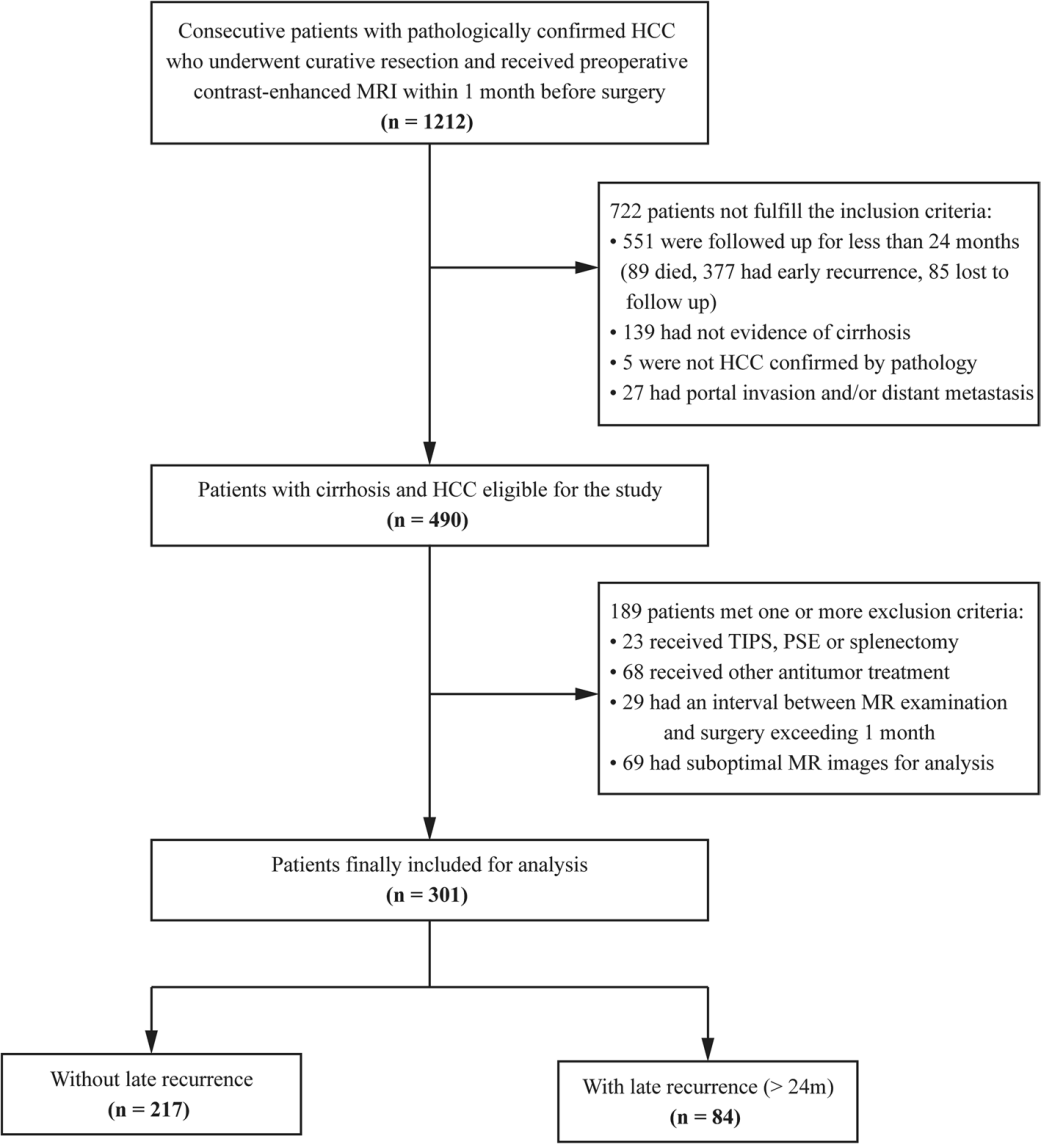
Flow diagram of the study population. HCC hepatocellular carcinoma, TIPS transjugular intrahepatic portosystemic shunt, PSE partial splenic embolization

As of May 2020, 301 eligible patients who were alive and free of early recurrence at 2 years after curative-intent resection were finally included (Fig. 1), with a mean age of 52.9 years (SD 10.6) and a sex distribution of 86.7% (261) males. The etiology of CLD was mostly related to hepatitis B virus infection (94.7%). Two hundred fourteen (71.1%) cases were categorized as BCLC stage A, with most having a single resectable tumor. The median interval between MRI examination and surgery was 8 days (IQR 3–8). Baseline characteristics of the study population are demonstrated in Table 1.

**Table 1:** Demographics and baseline characteristics of the study population

	**Entire cohort (*n* = 301)**	**Without late recurrence (*n* = 217)**	**With late recurrence (*n* = 84)**	***p* value**
**Demographics and clinical characteristics**				
Age (year)	52.9 (10.6)	52.9 (10.5)	52.9 (10.9)	0.989
Sex (Male)	261 (86.7%)	184 (84.8%)	77 (91.7%)	0.166
Etiology				0.394
Hepatitis B	285 (94.7%)	207 (95.4%)	78 (92.9%)	
Hepatitis C	11 (3.6%)	6 (2.8%)	5 (5.9%)	
Alcohol-related	5 (1.7%)	4 (1.8%)	1 (1.2%)	
Child–Pugh score	5.1 (0.5)	5.1 (0.3)	5.2 (0.4)	0.316
MELD score	7.5 (1.3)	7.4 (1.2)	7.8 (1.4)	0.010
ALBI score	− 2.89 (0.39)	− 2.92 (0.37)	− 2.83 (0.44)	0.090
FIB-4 index	3.39 (2.59)	3.02 (2.09)	4.33 (3.41)	0.001
aMAP score	59.1 (6.33)	58.5 (6.11)	60.6 (6.66)	0.013
APRI score	1.04 (0.90)	0.91 (0.79)	1.37 (1.09)	0.001
BCLC stage				0.095
0	61 (20.3%)	45 (20.7%)	16 (19.0%)	
A	214 (71.1%)	158 (72.8%)	56 (66.7%)	
B	26 (8.6%)	14 (6.5%)	12 (14.3%)	
**Laboratory parameters**				
AFP > 400 (ng/mL)	63 (21.1%)	43 (20.0%)	20 (23.8%)	0.570
Total bilirubin (mg/dL)	15.1 (6.6)	14.5 (6.4)	16.8 (6.8)	0.010
ALT (U/L)	47.4 (52.4)	43.6 (44.7)	57.2 (67.7)	0.092
AST (U/L)	41.5 (38.1)	38.5 (34.9)	49.5 (44.8)	0.044
Albumin (g/L)	42.9 (4.4)	43.1 (4.1)	42.5 (5.1)	0.344
Creatinine (mg/dL)	73.1 (13.3)	72.7 (13.4)	74.1 (13.2)	0.435
Prothrombin time (s)	12.1 (0.97)	12.0 (0.87)	12.5 (1.12)	0.001
Platelet count (× 10^9^/L)	123 (58.2)	128 (56.6)	109 (60.4)	0.012
**Radiological findings**				
Ascites (yes)	19 (6.3%)	12 (5.5%)	7 (8.3%)	0.527
Esophageal varices (yes)	168 (55.8%)	110 (50.7%)	58 (69.0%)	0.006
Splenomegaly (yes)	175 (58.1%)	117 (53.9%)	58 (69.0%)	0.024
Tumor number				0.020
1	268 (89.0%)	200 (92.2%)	68 (81.0%)	
2–3	27 (9.0%)	14 (6.4%)	13 (15.5%)	
> 3	6 (2.0%)	3 (1.4%)	3 (3.5%)	
Tumor size (cm)	3.78 (2.54)	3.82 (2.60)	3.68 (2.40)	0.661
**Pathological parameters**				
Tumor differentiation				0.810
High	84 (28.2%)	61 (28.5%)	23 (27.4%)	
Medium	199 (66.8%)	141 (65.9%)	58 (69.0%)	
Low	15 (5.0%)	12 (5.6%)	3 (3.6%)	
**Volumetric indices**				
Total liver volume (cm^3^)	1244 (296)	1253 (307)	1219 (266)	0.331
Left lobe volume (cm^3^)	422 (156)	431 (158)	400 (148)	0.112
Right lobe volume (cm^3^)	821 (242)	823 (254)	819 (211)	0.892
Caudate volume (cm^3^)	23 (15)	23 (16)	21 (11)	0.139
Spleen volume (cm^3^)	316 (157)	294 (134)	373 (194)	0.001

*MELD* model for end-stage liver disease, *ALBI* albumin-bilirubin, *BCLC* Barcelona Clinic Liver Cancer, *AFP* α-fetoprotein, *ALT* alanine transaminase, *AST* aspartate transaminase

During a median follow-up of 48.8 (IQR 36.6–65.8) months, 84 (27.9%) patients in the entire cohort developed late recurrence. The cumulative incidence of late recurrence at 3, 4, and 5 years was 11.0%, 19.2%, and 29.1%, respectively. Patients with and without late recurrence had similar age, sex distribution, and etiology of CLD, while patients with the outcome had more severe liver disease and worse liver function (Table 1).

### Predictive value of spleen volume

**Fig. 2:**
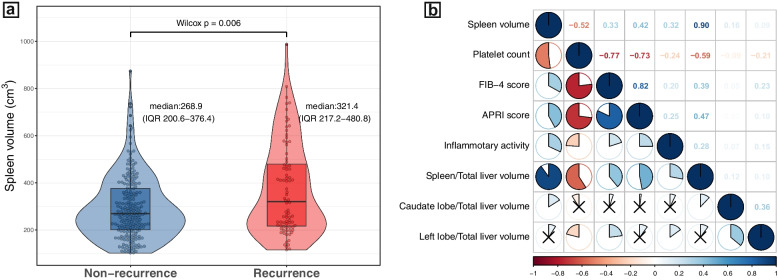
Analysis of spleen volume. **a** Distribution of spleen volume between the non-recurrence and recurrence groups. **b** Correlation between volumetric indices and laboratory/pathological parameters reflecting the severity of liver fibrosis/cirrhosis. Liver-related volumetric indices were calculated by excluding tumor volume. Black cross in each cell indicates a correlation *p* value > 0.05

Among the volumetric indices, only spleen volume was different between patients with (median = 321.4 cm^3^, IQR 217.2–480.8 cm^3^) and without late recurrence (median = 268.9 cm^3^, IQR 200.6–376.4 cm^3^, *p* = 0.006) (Fig. 2a). In addition, spleen volume showed moderate-to-poor correlation with laboratory and pathological parameters that reflect the severity of liver fibrosis and cirrhosis (*r* < 0.6 for all correlation), while no correlation was observed between other volumetric indices and laboratory/pathological parameters (*r* < 0.3 for all correlation) (Fig. 2b).

In univariable Cox regression analysis, variables associated with late recurrence (*p* < 0.1) were mostly related to the severity of underlying liver disease, except for tumor number and bilobar involvement of the liver (Table 2). For imaging features reflecting tumor aggressiveness, nonrim arterial phase hyperenhancement, nonenhancing capsule, and targetoid transitional or hepatobiliary phase appearance were the only significant predictors in univariable analysis (Table 3). In multivariable Cox analysis, only APRI score (HR 1.38, 95%CI 1.13–1.68, *p* = 0.001), tumor number (HR with 2–3 vs. 1: 2.02, 95%CI 1.15–3.65, *p* = 0.015; HR with > 3 vs. 1: 10.05, 95%CI 2.37–42.58, *p* < 0.001), and spleen volume (HR 1.01, 95%CI 1.00–1.01, *p* = 0.008) were associated with late recurrence, while no imaging features remained significant (Table 2).

**Table 2:** Uni- and multivariate Cox regression model for risk factors associated with late HCC recurrence

	**Univariable analysis**		**Multivariable analysis**	
**Variables**	**HR (95%CI)**	***p* value**	**HR (95%CI)**	***p* value**
Age (year)	1.00 (0.99–1.02)	0.641	…	…
Sex (male vs. female)	1.82 (0.83–4.01)	0.132	…	…
MELD score	1.23 (1.05–1.45)	0.010	…	…
FIB-4 index	1.14 (1.08–1.20)	< 0.001	…	…
aMAP score	1.06 (1.02–1.11)	0.005	…	…
APRI score	1.51 (1.29–1.78)	< 0.001	1.38 (1.13–1.68)	0.001
Total bilirubin (µmol/L)	1.04 (1.01–1.07)	0.011	…	…
Aspartate transaminase (U/L)	1.01 (1.00–1.01)	0.002	…	…
Prothrombin time (s)	1.39 (1.15–1.68)	< 0.001	…	…
Platelet count (× 10^9^/L)	0.99 (0.99–0.99)	0.007	…	…
Esophageal varices (yes vs. no)	1.87 (1.18–2.98)	0.008	…	…
Splenomegaly (yes vs. no)	1.99 (1.26–3.21)	0.003	…	…
Maximum tumor size (cm)	0.97 (0.88–1.07)	0.594	…	…
Tumor number				
2–3 vs. 1	1.95 (1.07–3.53)	0.028	2.05 (1.15–3.65)	0.015
> 3 vs. 1	8.45 (2.59–27.5)	< 0.001	10.05 (2.37–42.58)	0.002
Bilobar involvement	2.43 (1.21–4.87)	0.013	…	…
Satellite nodule (yes vs. no)^a^	1.62 (0.59–4.44)	0.345	…	…
MVI (yes vs. no)^a^	1.12 (0.54–2.30)	0.627	…	…
Tumor differentiation				
Moderate vs. well	1.22 (0.37–4.08)	0.743	…	…
Poor vs. well	1.58 (0.49–5.04)	0.443	…	…
Spleen volume (cm^3^)	2.02 (1.31–3.12)	0.001	1.01 (1.00–1.01)	0.008

*MELD* model for end-stage liver disease, *MVI* microvascular invasion, *HR* hazard ratio

^a^Presence of satellite nodule and MVI were determined by pathology

**Table 3:** Association between imaging features and late HCC recurrence in univariable Cox analysis and Fine-Gray competing risk analysis

Imaging features	Univariable Cox regression analysis		Univariable competing risk analysis	
	HR (95%CI)	*p* value	HR (95%CI)	*p* value
***LI-RADS major features***				
Nonrim arterial phase hyperenhancement	0.46 (0.21–0.99)	0.049	0.47 (0.20–1.10)	0.084
Nonperipheral “washout”	1.41 (0.82–2.44)	0.217	1.43 (0.83–2.46)	0.202
Enhancing “capsule”	0.74 (0.37–1.49)	0.405	0.76 (0.36–1.62)	0.480
Tumor size (cm)	1.03 (0.95–1.12)	0.482	1.01 (0.93–1.10)	0.762
***LI-RADS ancillary features***				
Nonenhancing “capsule”	5.65 (2.01–15.93)	0.001	5.80 (2.33–14.44)	< 0.001
Nodule-in-nodule architecture	0.88 (0.55–1.39)	0.576	0.83 (0.53–1.32)	0.435
Mosaic architecture	1.30 (0.77–2.20)	0.328	1.27 (0.76–2.14)	0.371
Blood products in mass	1.23 (0.75–2.01)	0.406	1.18 (0.72–1.93)	0.516
Fat in mass, more than adjacent liver	1.20 (0.77–1.87)	0.429	1.23 (0.80–1.91)	0.345
Mild-moderate T2 hyperintensity	0.67 (0.16–2.75)	0.578	1.03 (0.24–4.49)	0.975
Corona enhancement	0.93 (0.58–1.47)	0.747	0.88 (0.56–1.40)	0.602
Fat sparing in solid mass	0.83 (0.26–2.63)	0.751	0.88 (0.27–2.91)	0.835
Iron sparing in solid mass	1.30 (0.67–2.52)	0.441	1.16 (0.57–2.34)	0.692
TP hypointensity^a^	0.74 (0.09–5.84)	0.774	0.74 (0.14–4.02)	0.723
Marked HBP hypointensity^a^	0.52 (0.17–1.56)	0.241	0.52 (0.18–1.49)	0.229
***LR-M features***				
Rim arterial phase hyperenhancement	0.48 (0.07–3.43)	0.463	0.44 (0.06–3.41)	0.439
Peripheral “washout”	1.50 (0.21–10.84)	0.685	1.48 (0.15–14.91)	0.740
Targetoid restriction	0.70 (0.17–2.89)	0.626	0.71 (0.14–3.57)	0.687
Targetoid TP or HBP appearance^a^	12.52 (2.41–64.9)	0.003	12.52 (3.13–49.9)	< 0.001
Marked diffusion restriction	0.76 (0.40–1.43)	0.394	0.81 (0.42–1.56)	0.536
Infiltrative appearance	1.66 (0.61–4.55)	0.322	1.25 (0.46–3.42)	0.665
Necrosis or severe ischemia	0.94 (0.58–1.52)	0.805	0.83 (0.51–1.35)	0.450
***LI-RADS category***				
LR-4	Ref	…	Ref	…
LR-5	0.79 (0.41–1.54)	0.496	0.77 (0.39–1.53)	0.468
LR-M	0.63 (0.19–2.01)	0.434	0.65 (0.18–2.37)	0.512
***Other tumor-related prognostic features***				
≥ 50% arterial phase hyperenhancement	0.87 (0.51–1.47)	0.605	0.93 (0.54–1.60)	0.781
Mild-moderate T2 peritumoral hyperintensity	0.80 (0.38–1.67)	0.550	0.77 (0.36–1.67)	0.516
PVP peritumoral hypoenhancement	1.85 (1.02–3.38)	0.044	1.66 (0.91–3.05)	0.105
HBP peritumoral hypointensity^a^	2.03 (0.66–6.26)	0.219	2.03 (0.70–5.91)	0.203
Markedly low ADC value	0.59 (0.22–1.62)	0.307	0.60 (0.21–1.73)	0.358
Complete capsule	0.80 (0.49–1.30)	0.367	0.81 (0.50–1.33)	0.406
Non-smooth tumor margin	1.01 (0.62–1.61)	0.990	0.99 (0.62–1.58)	0.966
Intratumoral artery	1.15 (0.63–2.09)	0.648	1.07 (0.60–1.93)	0.810
Parallels blood pool enhancement	2.56 (0.35–18.47)	0.352	2.77 (2.15–3.56)	< 0.001
Satellite nodule	1.72 (0.63–4.71)	0.291	1.24 (0.42–3.67)	0.701
Tumor growth subtype				
Single nodule	Ref	…	Ref	…
Single nodule with extranodular growth	0.89 (0.57–1.38)	0.599	0.91 (0.59–1.42)	0.690
Confluent multinodular or infiltrative type	4.91 (0.64–37.49)	0.125	3.86 (0.95–7.86)	0.902

Hazard ratio (95%CI) was calculated by comparing the presence versus absence of the corresponding imaging feature except for the LI-RADS category and tumor growth subtype

*TP* transitional phase, *HBP* hepatobiliary phase, *PVP* portal venous phase, *ADC* apparent diffusion coefficient, *HR* hazard ratio

^a^For imaging features achieved from TP or HBP, gadoxetate acid disodium was used in only 46 patients

**Fig. 3:**
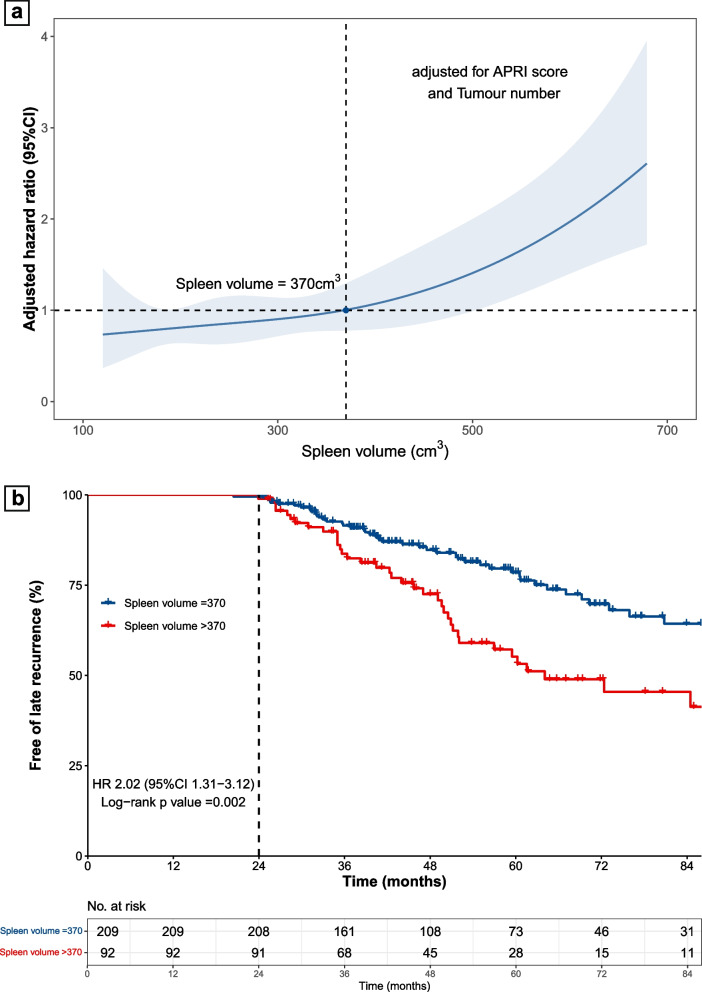
Predictive value of spleen volume. **a** Non-linear correlation between spleen volume and the risk of late recurrence. **b** Cumulative rate of late recurrence between high spleen volume (> 370 cm^3^) and low spleen volume (≤ 370 cm.^3^)

Afterwards, a restricted cubic spline was used to demonstrate the correlation between spleen volume and risk of late recurrence (Fig. 3a). The risk of late recurrence was steady and relatively low in patients with a baseline spleen volume lower than 370 cm^3^, but increased progressively to the increase in spleen volume in those with a volume higher than 370 cm^3^. Thus, using 370 cm^3^ as the cutoff value, baseline spleen volume could effectively identify a subgroup of patients with a substantially higher risk of late recurrence (HR 2.02, 95%CI 1.31–3.12, *p* = 0.002) (Fig. 3b). Furthermore, higher baseline spleen volume was also associated with substantially worse RFS and OS after resection (Supplementary Fig. 2).

In additional subgroup analysis, the effect of spleen volume on late recurrence was more pronounced in patients with lower BCLC stage, maximum tumor size > 5 cm, and solitary and well-differentiated tumor, though the differences between subgroups were not significant (*p* for interaction > 0.05 for all comparisons) (Supplementary Fig. 3).

### Development of a spleen volume-based prediction score

Using 5-year recurrence as the endpoint, a spleen-based risk score was developed by fitting a Cox regression model, with APRI score, spleen volume (fitted as non-linearity), and tumor number as components.

**Fig. 4:**
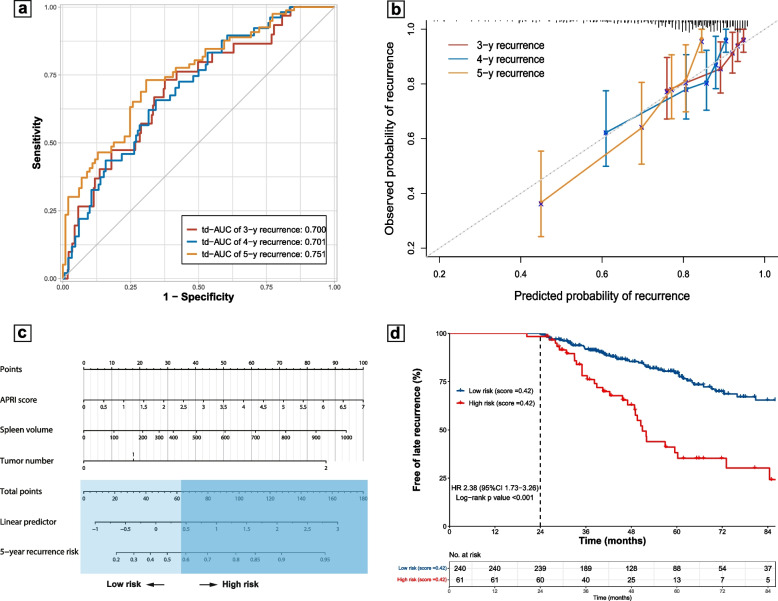
Development and validation of a risk prediction score. **a** Time-dependent area under the curve (td-AUC) of the risk score in predicting 3-, 4-, and 5-year recurrence risk. **b** Calibration plot of the risk score. **c** Nomogram for predicting late recurrence risk by incorporating APRI score, spleen volume, and tumor number as predictors. **d** Cumulative rate of late recurrence stratified according to the risk groups defined by the risk score (patients with a score > 0.42 were defined as high-risk)

The risk score showed a td-AUC of 0.700 at 3 years, 0.701 at 4 years, and 0.751 at 5 years, and these measures were 0.693, 0.700, and 0.748 after optimism-correction in the internal validation, indicating minimal overfitting of the developed score (Fig. 4a). Calibration plot indicated good consistency between the predicted and observed endpoint, and the Brier score confirmed that the developed score was well calibrated (Brier score at 3, 4, 5 years: 9.7, 15.0, 17.2; optimism-corrected Brier score at 3, 4, 5 years: 9.9, 15.3, 17.7) (Fig. 4b). A nomogram was then generated to facilitate clinical adoptions (Fig. 4c).

To identify a high-risk group, the final score was split at the 80th percentile (= 0.42). Using this cutoff, the final score could effectively stratify the entire cohort into two risk groups, with the high-risk group demonstrating a markedly higher risk of late recurrence compared with the low-risk group (3-, 4-, 5-year recurrence risk: 23.9%, 39.7%, 64.7% vs. 8.2%, 14.6%, 20.5%; *p* < 0.001 for each survival rate) (Fig. 4d).

### Pattern of late recurrence

Among those who developed late HCC recurrence, 26 patients had intrahepatic local recurrence (ILR), 65 had intrahepatic distant recurrence (IDR), and 13 had extrahepatic metastasis (EM) (Supplementary Table 1). The risk score showed higher discriminative ability in predicting IDR (td-AUC = 0.790) compared with ILR (0.704) and EM (0.753), and the high-risk group had a higher incidence of IDR (44.3% vs. 15.8%) and EM (11.5% vs. 2.5%) compared with the low-risk group. In addition, density curves showed that IDR and EM in the high-risk group chronologically preceded that in the low-risk group, suggesting a more intensive recurrence in the high-risk group at the early stage after resection (Supplementary Fig. 4).

### Sensitivity analysis

In the first sensitivity analysis that used death (over 2 years) as a competing event, predictors of late recurrence in the uni- and multivariable Fine and Gray competing risk analysis remained similar as in the Cox regression analysis (Table 3 and Supplementary Table 2). In the second analysis, no marked improvement in the predictive accuracy was observed after incorporating postoperative (i.e., surgical or pathological) parameters (Supplementary Table 3).

## Discussion

Late recurrence of HCC after curative-intent resection is widely regarded as de novo multicentric tumors that are mostly related to the severity of the underlying liver disease. The present study found that preoperative spleen volume, which could be quickly and accurately measured using MRI and AI techniques, was independently associated with an increased risk of late recurrence and decreased survival in patients with cirrhosis. A risk score based on the APRI score, spleen volume, and tumor number was developed and internally validated, demonstrating good risk prediction and stratification ability.

Unlike early HCC recurrence of which the prevalence and risk factors have been well recognized, data are sparse with regard to late recurrence. A prospective study reported that 27 out of 87 (31.0%) patients with HCC and CLD developed late recurrence after liver resection [12]. Another large-scale multicenter study based on an HBV-predominant cohort reported an incidence of 41.3% during a median follow-up of 78.0 months [19]. The late recurrence rate was comparatively lower in our cohort (27.8%), which might be attributed to the higher percentage of early HCC stage we included (91.4% of patients were at BCLC-0/A and no BCLC-C were included). Of note, since cirrhosis is a well-established risk factor, this study only included patients with confirmed cirrhosis, so as to evaluate the incremental value of volumetric indices and MRI imaging features for late recurrence prediction.

The role of liver and spleen-related indices in predicting key events (e.g., occurrence of decompensation and HCC) during the natural history of CLD has been adequately addressed. Previous results found that liver stiffness was predictive of HCC recurrence after resection [20–22]. More recently, however, spleen stiffness instead of liver stiffness was suggested as the only independent predictor of postoperative late recurrence [12]. In addition to stiffness change, morphological change of the spleen has also been associated with the severity of liver fibrosis [13, 14, 23], as progression in fibrosis results in a reduction in liver volume and increase in spleen volume. Besides, compared with a diameter measured on cross-sectional or craniocaudal image, three-dimensional spleen volume can reflect the complex morphology more accurately and thereby had superior predictive ability for decompensation [24].

Therefore, we hypothesized that spleen volume could be a quantitative parameter to reflect the severity of cirrhosis and predict the risk of late HCC recurrence. We found that preoperative spleen volume was independently associated with late recurrence, and a cutoff value of 370 cm^3^ was determined to identify patients with an increased risk of late recurrence, which was slightly higher than the suggested population-based reference (322 cm^3^) [25]. Yoo et. al [26]. proposed a cutoff of 532 mL to predict HCC occurrence in patients with compensated CLD, which was significantly higher than our determined cutoff. This difference may be attributed to the heterogeneity between HCC occurrence and late recurrence, as the pathogenesis of the latter includes multiple factors besides cirrhosis. Specifically, tumor number was identified as another independent predictor of late recurrence, which has also been observed previously [9, 27, 28]. Interestingly, tumor number was the only tumor-related parameter associated with late recurrence, whereas other imaging features or pathological indices reflecting tumor aggressiveness were not associated with the outcome. These results further confirmed that late HCC recurrence represents a distinct pattern of hepatocarcinogenesis compared with early recurrence and is more likely driven by the severity of underlying liver disease.

CT and MRI are the recommended procedures for HCC screening and postoperative surveillance [5]. Compared with CT, MRI has the advantage in providing information on tumor aggressiveness via multiparametric sequences [29, 30], allowing for simultaneously evaluating the risk of early and late recurrence. Besides, the main problem that hinders the widespread use of spleen volume is the time-consuming segmentation process and interobserver variation. With the advancements in the field of AI techniques, spleen segmentation and volume measurement can now be accomplished in a fully automated, time-efficient, and reproducible manner, making it possible to be integrated into routine clinical workflows.

Because of the heterogeneous risk of late HCC recurrence, we proposed a prognostic score to estimate the risk of late recurrence to inform individualized clinical decision-making. Specifically, patients categorized as high-risk may benefit from more regular and intensive surveillance (e.g., shorter follow-up interval and a preference for MRI over CT) [19]. Moreover, intrahepatic recurrence (both local and distant) occurred earlier in the high-risk group, and most patients with extrahepatic metastasis had simultaneous or previous intrahepatic recurrence, underscoring the need for establishing a risk score-guided surveillance strategy.

Several limitations exist in our study. First, selection and indication bias are inherent to the retrospective design. Second, as a retrospective study we were unable to obtain information on HBsAg seroclearance for patients with HBV infection and sustained virological response for patients with HCV infection, which were suggested to decrease recurrence risk, though the results remain controversial [31, 32]. Third, spleen volume was not directly compared with liver and spleen stiffness in our study. Fourth, different scanning protocols used might increase data variability and reduce results reliability. Lastly, the relatively small number of patients included limits the ability to draw firm conclusions relative to the general population, and multicenter prospective studies are warranted to validate and refine our results.

In conclusion, preoperative spleen volume measured on MRI with AI techniques is a reliable and sensitive parameter in predicting late recurrence risk after curative-intent resection in patients with HCC and established cirrhosis. A risk score based on spleen volume was developed, providing the opportunity for individualized risk stratification and tailoring of postoperative surveillance strategy.

### Supplementary Information

**Additional file 1: Supplementary materials. **Supplementary tables. Supplementary figures.

## Data Availability

The datasets used and/or analyzed during the current study are available from the corresponding author upon reasonable request.

## Abbreviations

AI: Artificial intelligence
AUC: Area under the curve
BCLC: Barcelona Clinic Liver Cancer
CI: Confidence interval
CLD: Chronic liver disease
EM: Extrahepatic metastasis
HCC: Hepatocellular carcinoma
HR: Hazard ratio
IDR: Intrahepatic distant recurrence
ILR: Intrahepatic local recurrence
LI-RADS: Liver Imaging Reporting and Data System
OS: Overall survival
RFS: Recurrence-free survival

## References

[1] Kulik L, El-Serag HB (2019). Epidemiology and management of hepatocellular carcinoma. Gastroenterology.

[2] Villanueva A (2019). Hepatocellular carcinoma. N Engl J Med.

[3] Yang JD, Hainaut P, Gores GJ (2019). A global view of hepatocellular carcinoma: trends, risk, prevention and management. Nat Rev Gastroenterol Hepatol.

[4] Sung H, Ferlay J, Siegel RL (2021). Global cancer statistics 2020: GLOBOCAN estimates of incidence and mortality worldwide for 36 cancers in 185 countries. CA Cancer J Clin.

[5] EASL Clinical Practice Guidelines (2018). Management of hepatocellular carcinoma. J Hepatol.

[6] Heimbach JK, Kulik LM, Finn RS (2018). AASLD guidelines for the treatment of hepatocellular carcinoma. Hepatology.

[7] Gluer AM, Cocco N, Laurence JM (2012). Systematic review of actual 10-year survival following resection for hepatocellular carcinoma. HPB (Oxford).

[8] Maluccio M, Covey A (2012). Recent progress in understanding, diagnosing, and treating hepatocellular carcinoma. CA Cancer J Clin.

[9] Imamura H, Matsuyama Y, Tanaka E (2003). Risk factors contributing to early and late phase intrahepatic recurrence of hepatocellular carcinoma after hepatectomy. J Hepatol.

[10] Poon RT, Fan ST, Ng IO (2000). Different risk factors and prognosis for early and late intrahepatic recurrence after resection of hepatocellular carcinoma. Cancer.

[11] Portolani N, Coniglio A, Ghidoni S (2006). Early and late recurrence after liver resection for hepatocellular carcinoma: prognostic and therapeutic implications. Ann Surg.

[12] Marasco G, Colecchia A, Colli A (2019). Role of liver and spleen stiffness in predicting the recurrence of hepatocellular carcinoma after resection. J Hepatol.

[13] Son JH, Lee SS, Lee Y (2020). Assessment of liver fibrosis severity using computed tomography-based liver and spleen volumetric indices in patients with chronic liver disease. Eur Radiol.

[14] Kwon JH, Lee SS, Yoon JS (2021). Liver-to-spleen volume ratio automatically measured on CT predicts decompensation in patients with B viral compensated cirrhosis. Korean J Radiol.

[15] Roberts LR, Sirlin CB, Zaiem F (2018). Imaging for the diagnosis of hepatocellular carcinoma: A systematic review and meta-analysis. Hepatology.

[16] Collins GS, Reitsma JB, Altman DG (2015). Transparent reporting of a multivariable prediction model for individual prognosis or diagnosis (TRIPOD): the TRIPOD statement. BMJ.

[17] Scheuer PJ (1991). Classification of chronic viral hepatitis: a need for reassessment. J Hepatol.

[18] Ishak K, Baptista A, Bianchi L (1995). Histological grading and staging of chronic hepatitis. J Hepatol.

[19] Xu XF, Xing H, Han J (2019). Risk factors, patterns, and outcomes of late recurrence after liver resection for hepatocellular carcinoma: a multicenter study from China. JAMA Surg.

[20] Jung KS, Kim SU, Choi GH (2012). Prediction of recurrence after curative resection of hepatocellular carcinoma using liver stiffness measurement (FibroScan®). Ann Surg Oncol.

[21] Jung KS, Kim JH, Kim SU (2014). Liver stiffness value-based risk estimation of late recurrence after curative resection of hepatocellular carcinoma: development and validation of a predictive model. PLoS One.

[22] Zhang L, Chen J, Jiang H (2022). MR elastography as a biomarker for prediction of early and late recurrence in HBV-related hepatocellular carcinoma patients before hepatectomy. Eur J Radiol.

[23] Tan BG, Tang Z, Ou J, et al (2022) A novel model based on liver/spleen volumes and portal vein diameter on MRI to predict variceal bleeding in HBV cirrhosis. Eur Radiol 10.1007/s00330-022-09107-5 36048206

[24] Yu Q, Xu C, Li Q (2022). Spleen volume-based non-invasive tool for predicting hepatic decompensation in people with compensated cirrhosis (CHESS1701). JHEP Rep.

[25] Kim DW, Ha J, Lee SS (2021). Population-based and personalized reference intervals for liver and spleen volumes in healthy individuals and those with viral hepatitis. Radiology.

[26] Yoo J, Kim SW, Lee DH (2021). Prognostic role of spleen volume measurement using computed tomography in patients with compensated chronic liver disease from hepatitis B viral infection. Eur Radiol.

[27] Dai T, Deng M, Ye L (2021). Nomograms based on clinicopathological factors and inflammatory indicators for prediction of early and late recurrence of hepatocellular carcinoma after surgical resection for patients with chronic hepatitis B. Ann Transl Med.

[28] Yang Y, Chen Y, Ye F (2021). Late recurrence of hepatocellular carcinoma after radiofrequency ablation: a multicenter study of risk factors, patterns, and survival. Eur Radiol.

[29] Lee YJ, Lee JM, Lee JS (2015). Hepatocellular carcinoma: diagnostic performance of multidetector CT and MR imaging-a systematic review and meta-analysis. Radiology.

[30] Liu X, Jiang H, Chen J (2017). Gadoxetic acid disodium-enhanced magnetic resonance imaging outperformed multidetector computed tomography in diagnosing small hepatocellular carcinoma: a meta-analysis. Liver Transpl.

[31] Sapena V, Enea M, Torres F (2022). Hepatocellular carcinoma recurrence after direct-acting antiviral therapy: an individual patient data meta-analysis. Gut.

[32] Yoo S, Kim JY, Lim YS (2022). Impact of HBsAg seroclearance on late recurrence of hepatitis B virus-related hepatocellular carcinoma after surgical resection. J Hepatol.

