# Supplementary material for: Prediction of late recurrence after curative-intent resection using MRI-measured spleen volume in patients with hepatocellular carcinoma and cirrhosis
Source: Insights Imaging. 2024 Feb 2;15:31. doi: 10.1186/s13244-024-01609-8 (PMC10834928; doi:10.1186/s13244-024-01609-8)
Supplement: Supplementary file 1 — Additional file 1: Supplementary materials. Supplementary tables. Supplementary figures. [file 13244_2024_1609_MOESM1_ESM.docx]

**Prediction of late recurrence after curative-intent resection using MRI-measured spleen volume in patients with hepatocellular carcinoma and cirrhosis**

**ELECTRONIC SUPPLEMENTARY MATERIAL**

**TABLE OF CONTENTS**

- **MRI acquisition protocol**
- **Definition and diagnosis of different patterns of late recurrence**
- **Supplementary table 1.** Details of late recurrence pattern in the entire cohort and in the low- and high-risk group stratified by the final score.
- **Supplementary table 2.** Uni- and multivariable Fine and Gray competing risk analyses for risk factors associated with late HCC recurrence.
- **Supplementary table 3.** Predictive accuracy of different model combinations based on the final risk score and postoperative parameters.
- **Supplementary figure 1.** Fully-automated segmentation and calculation of liver and spleen volume using SenseCare software.
- **Supplementary figure 2.** Kaplan-Meier curves of (**A**) recurrence-free survival, and (**B**) overall survival between two risk groups stratified according to baseline spleen volume (>370 vs. ≤370 cm^3^).
- **Supplementary figure 3.** Forest plot demonstrating the effect of spleen volume on late recurrence in pre-specified subgroups.
- **Supplementary figure 4.** Prognostic value of the risk score in predicting different recurrence patterns.

**MRI acquisition protocol**

All enhanced MR images were acquired on one of the following eight types of 3.0 T or 1.5 T systems (Siemens MAGNETOM Skyra; Siemens TrioTim; Siemens Avanto; GE SIGNA™ Architect; GE Discovery MR 750; GE SIGNA™ Premier; Philips Ingenia Elition X; uMR588). The MRI sequences included: (***a***) T2-weighted imaging; (***b***) diffusion-weighted imaging (b values: 0, 50, 500, 800, 1000, and 1200 s/mm^2^ [Siemens MAGNETOM Skyra]; 0, 50, 800 s/mm^2^ [Siemens TrioTim; Siemens Avanto]; 0, 50, 1000 s/mm^2^ [Siemens Avanto; GE SIGNA™ Architect; uMR588]; 0, 50, 1000 s/mm^2^ [GE SIGNA™ Architect; GE SIGNA™ Premier; uMR588]; 0, 200, 800, and 1000 s/mm^2^ [GE Discovery MR 750]; 0, 200, 1000 s/mm^2^ [Philips Ingenia Elition X]) with apparent diffusion coefficient maps reconstructed using the monoexponential model; (***c***) in- and opposed-phase T1-weighted imaging. For extracellular contrast agent (ECA)-enhanced MR images, dynamic T1-weighted imaging before and after injection of contrast agent in the late arterial phase, portal venous phase (60s after start of contrast media injection), and delayed phase (180s after injection) were obtained. 0.1 mmol/kg of gadopentetate dimeglumine (Magnevist®; Bayer Schering Pharma AG, Berlin, Germany) or gadoterate meglumine (Dotarem®; Guerbet, Paris, France) or gadobenate dimeglumine (MultiHance®; Bracco, Shanghai, China) was injected at a rate of 2.5 ml/s. For gadoxetate disodium (EOB)-enhanced MR images, dynamic T1-weighted imaging before and after injection of EOB in the late arterial phase, portal venous phase (60s after injection), transitional phase (180s after injection), and hepatobiliary phase (20min after injection) were obtained. 0.025 mmol/kg of EOB (Primovist®; Bayer Schering Pharma AG, Berlin, Germany) was injected at a rate of 1.0-2.0 ml/s, with an immediately followed 20-30 ml saline ﬂush. The arterial phase images were achieved either with acquisition triggered 7s after arrival of the contrast bolus in the celiac trunk or a multiple arterial phase (MAP) imaging technique.

**MR Sequences and Parameters.**

| **Sequence** | **Fat suppression** | **TR**  **(ms)** | **TE**  **(ms)** | **Flip angle (°)** | **ST**  **(mm)** | **Spacing**  **(mm)** | **Matrix**  **size** | **FOV**  **(mm^2^)** | **Acquisition Time (s)** |
| --- | --- | --- | --- | --- | --- | --- | --- | --- | --- |
| **Siemens MAGNETOM Skyra 3.0 Tesla (18-channel body array coil)** | | | | | | | | | |
| T2-weighted 2D FSE | Yes | 2160 | 100 | 160 | 6 | 1.8 | 320×288 | 433×433 | 36 |
| Diffusion-weighted imaging* | Yes | 5600 | 68 | 90 | 6 | 1.8 | 100×76 | 380×289 | 233 |
| In- and opposed-phase T1-weighted imaging | No | 81 | 2.72/1.4 | 70 | 6 | 1.8 | 352×286 | 400×325 | 24 |
| Dynamic T1-weighted 3D GRE | Yes | 3.95 | 1.92 | 9 | 2.5 | - | 352×256 | 400×296 | 14 |
| **Siemens TrioTim 3.0 Tesla (8-channel body anterior coil)** | | | | | | | | | |
| T2-weighted 2D FSE | Yes | 2700 | 95 | 140 | 6 | 7.8 | 320×147 | 442×254 | Respiratory gating |
| Diffusion-weighted imaging* | Yes | 5900 | 76 | 90 | 6 | 7.8 | 192×154 | 393×393 | 245 |
| In- and opposed-phase T1-weighted imaging | No | 181 | 2.2 | 65 | 6 | 7.8 | 256×131 | 410×269 | 18 |
| Dynamic T1-weighted 3D GRE | Yes | 3.47 | 1.25 | 9 | 2.4 | - | 320×133 | 434×257 | 17 |
| **Siemens Avanto 1.5 Tesla (30-channel body anterior coil)** | | | | | | | | | |
| T2-weighted 2D FSE | Yes | 2530 | 84 | 150 | 6 | 7.8 | 256×187 | 293×251 | 47 |
| Diffusion-weighted imaging* | Yes | 3600 | 88 | 90 | 6 | 7.8 | 192×115 | 310×232 | 92 |
| In- and opposed-phase T1-weighted imaging | No | 72 | 2.22 | 70 | 6 | 7.8 | 256×158 | 328×225 | 16 |
| Dynamic T1-weighted 3D GRE | Yes | 5.41 | 2.39 | 10 | 2.5 | - | 320×138 | 382×238 | 15 |
| **GE SIGNA™ Architect 3.0 Tesla (30-channel body anterior coil)** | | | | | | | | | |
| T2-weighted 2D FSE | Yes | 2400 | 85 | 111 | 7 | 2 | 320×192 | 380×304 | 34 |
| Diffusion-weighted imaging* | Yes | 5000 | Minimum | 90 | 7 | 2 | 160×128 | 380×342 | Respiratory gating |
| In- and opposed-phase T1-weighted imaging | No | 233.8 | 2.3/1.1 | 55 | 7 | 2 | 160×288 | 380×323 | 18 |
| Dynamic T1-weighted 3D GRE | Yes | 3.9 | 1.7 | 15 | 3 | - | 320×240 | 380×380 | 15 |
| **GE SIGNA™ Premier 3.0 Tesla (30-channel body anterior coil)** | | | | | | | | | |
| T2-weighted 2D FSE | Yes | 2200 | 85 | 111 | 7 | 2 | 320×224 | 304×380 | 47 |
| Diffusion-weighted imaging* | Yes | 5000 | Minimum | 90 | 7 | 2 | 120 × 240 | 380× 380 | Respiratory gating |
| In- and opposed-phase T1-weighted imaging | No | 146.8 | 2.3/1.1 | 55 | 7 | 2 | 320×192 | 342×380 | 16 |
| Dynamic T1-weighted 3D GRE | Yes | 3.2 | 1.4 | 15 | 2.4 | - | 320×240 | 380× 380 | 15 |
| **GE Discovery MR 750 3.0 Tesla (16-channel phased-array torsor coil)** | | | | | | | | | |
| T2-weighted 2D FSE | Yes | 6315 | 78 | 111 | 6 | 2 | 288×244 | 360×280 | Respiratory gating |
| Diffusion-weighted imaging* | Yes | 9230 | Minimum | 90 | 6 | 2 | 128 × 128 | 360× 380 | Respiratory gating |
| In- and opposed-phase T1-weighted imaging | No | 150 | 2.5/1.3 | 70 | 6 | 2 | 288×192 | 420×420 | 31 |
| Dynamic T1-weighted 3D GRE | Yes | 4.1 | 1.9 | 15 | 2 | - | 512×512 | 380× 300 | 15 |
| **Philips Ingenia Elition X 3.0 Tesla (16-channel body anterior coil)** | | | | | | | | | |
| T2-weighted 2D FSE | Yes | 1883.51 | 90 | 90 | 6.8 | 8.5 | 272×78 | 239×69 | 46 |
| Diffusion-weighted imaging* | Yes | 1653.65 | 60.29 | 90 | 7 | 8.5 | 142×140 | 141×139 | 52 |
| In- and opposed-phase T1-weighted imaging | No | 164.53 | 1.15 | 50 | 6 | 7.5 | 256×201 | 206×162 | 11 |
| Dynamic T1-weighted 3D GRE | Yes | 4.20 | 0.00 | 10 | 3 | 1.5 | 344×252 | 303×222 | 13 |
| **uMR588 1.5 Tesla (6-channel body anterior coil)** | | | | | | | | | |
| T2-weighted 2D FSE | Yes | 2600 | 99.2 | 90 | 6.5 | 1.5 | 256×168 | 427×320 | 39 |
| Diffusion-weighted imaging* | Yes | 3350 | 77 | 90 | 6.5 | 10 | 128×92 | 320×400 | Respiratory gating |
| In- and opposed-phase T1-weighted imaging | No | 117.6 | 2.27 | 60 | 6.5 | 1.3 | 256×174 | 320×400 | 29 |
| Dynamic T1-weighted 3D GRE | Yes | 4.2 | 1.88 | 10 | 2.5 | - | 256×154 | 255×400 | 13 |

Note. —TR = repetition time; TE = echo time; ST = section thickness; FOV = field of view; 2D = two-dimensional; 3D = three-dimensional; FSE = fast spin-echo; GRE = gradient recall echo. * Images were acquired under free breath.

**Definition and diagnosis of different patterns of late recurrence**

Three patterns of late HCC recurrence were diagnosed and recorded during follow-up based on imaging findings (ultrasound, CT or MRI): **(a)** intrahepatic local recurrence (ILR), defined as the identification of tumor foci at the edge of the surgical regions, **(b)** intrahepatic distant recurrence (IDR), defined as the identification of new intrahepatic tumor within liver subsegments different from the surgical regions or in the same liver subsegment that was not adjacent to the surgical regions, and **(c)** extrahepatic metastasis (EM), defined as the identification of new metastasis outside the liver.

**Supplementary table 1.** Details of late recurrence pattern in the entire cohort and in the low- and high-risk group stratified by the final score.

|  | **Entire cohort**  **(N=301)** | **Low-risk group**  **(n=240)** | **High-risk group**  **(n=61)** | **P value** |
| --- | --- | --- | --- | --- |
| **Recurrence** | | | | <0.001 |
| No | 217 (72.1%) | 188 (78.3%) | 29 (47.5%) |  |
| Yes | 84 (27.9%) | 52 (21.7%) | 32 (52.5%) |  |
| **Intrahepatic local recurrence** | | | | 0.255 |
| No | 275 (91.4%) | 222 (92.5%) | 53 (86.9%) |  |
| Yes | 26 (8.6%) | 18 (7.5%) | 8 (13.1%) |  |
| **Intrahepatic distant recurrence** | | | | <0.001 |
| No | 236 (78.4%) | 202 (84.2%) | 34 (55.7%) |  |
| Yes | 65 (21.6%) | 38 (15.8%) | 27 (44.3%) |  |
| **Extrahepatic metastasis** | | | | 0.006 |
| No | 288 (95.7%) | 234 (97.5%) | 54 (88.5%) |  |
| Yes | 13 (4.3%) | 6 (2.5%) | 7 (11.5%) |  |

Pattern of late recurrence was divided into three types: intrahepatic local recurrence (ILR), intrahepatic distant recurrence (IDR) and extrahepatic metastasis (EM). For patients who had simultaneous two or more types at the initial diagnosis, each type was counted separately.

**Supplementary table 2.** Uni- and multivariable Fine and Gray competing risk analyses for risk factors associated with late HCC recurrence.

|  | **Univariable analysis** | |  | **Multivariable analysis** | |
| --- | --- | --- | --- | --- | --- |
| **Variables** | **sHR (95%CI)** | ***P* value** |  | **sHR (95%CI)** | ***P* value** |
| Age (per year) | 1.00 (0.98-1.02) | 0.710 |  | … | … |
| Sex (male vs. female) | 1.96 (0.93-3.98) | 0.074 |  | … | … |
| MELD score | 1.23 (1.04-1.45) | 0.016 |  | … | … |
| FIB-4 index | 1.13 (1.06-1.20) | <0.001 |  | … | … |
| aMAP score | 1.05 (1.01-1.09) | 0.020 |  | … | … |
| APRI score | 1.47 (1.28-1.69) | <0.001 |  | 1.26 (1.06-1.50) | 0.008 |
| Total bilirubin | 1.03 (1.01-1.06) | 0.017 |  | … | … |
| Aspartate transaminase | 1.01 (1.00-1.01) | <0.001 |  | … | … |
| Prothrombin time | 1.40 (1.10-1.78) | 0.006 |  | … | … |
| Platelet count | 0.99 (0.99-1.00) | 0.063 |  | … | … |
| Esophageal varices (yes vs. no) | 1.98 (1.25-3.12) | 0.003 |  | … | … |
| Splenomegaly (yes vs. no) | 1.91 (1.21-3.01) | 0.005 |  | … | … |
| Tumor number |  |  |  |  |  |
| 2-3 vs. 1 | 2.10 (1.23-3.58) | 0.007 |  | 2.09 (1.13-3.88) | 0.018 |
| >3 vs. 1 | 3.28 (0.89-12.1) | 0.074 |  | 2.63 (0.57-12.2) | 0.220 |
| Maximum tumor size (per cm) | 0.98 (0.89-1.08) | 0.630 |  | … | … |
| Satellite nodule (yes vs. no) | 1.60 (0.59-4.33) | 0.350 |  | … | … |
| MVI (yes vs. no) | 1.13 (0.55-2.41) | 0.740 |  | … | … |
| Tumor differentiation |  |  |  |  |  |
| Medium vs. high | 1.99 (0.64-6.23) | 0.240 |  | … | … |
| Low vs. high | 1.60 (0.47-5.39) | 0.450 |  | … | … |
| Spleen volume | 1.01 (1.00-1.01) | <0.001 |  | 1.00 (1.00-1.01) | 0.012 |

sHR subdistribution hazard ratio, MELD model for end-stage liver disease, APRI aspartate aminotransferase to platelet ratio index, MVI microvascular invasion

**Supplementary table 3.** Predictive accuracy of different model combinations based on the final risk score and postoperative parameters.

|  | **Time-dependent AUC** | | |  | **Brier score** | | |
| --- | --- | --- | --- | --- | --- | --- | --- |
| **Model combination** | 3-year | 4-year | 5-year |  | 3-year | 4-year | 5-year |
| Risk score (*n* = 153) | 0.780 | 0.817 | 0.778 |  | 12.0 | 16.3 | 18.2 |
| Risk score + MVI | 0.785 | 0.820 | 0.777 |  | 12.0 | 16.3 | 18.4 |
| Risk score (*n* = 134) | 0.750 | 0.780 | 0.786 |  | 11.9 | 15.6 | 14.7 |
| Risk score + Satellite nodule | 0.750 | 0.789 | 0.793 |  | 11.9 | 16.0 | 14.9 |
| Risk score (*n* = 280) | 0.709 | 0.704 | 0.749 |  | 9.7 | 14.6 | 16.9 |
| Risk score + Capsular involvement | 0.703 | 0.689 | 0.752 |  | 9.7 | 14.7 | 16.6 |
| Risk score (*n* = 271) | 0.685 | 0.705 | 0.745 |  | 9.2 | 14.3 | 16.5 |
| Risk score + Transfusion | 0.696 | 0.714 | 0.743 |  | 9.1 | 14.2 | 16.5 |
| Risk score (*n* = 296) | 0.700 | 0.701 | 0.751 |  | 9.6 | 14.9 | 17.2 |
| Risk score + Tumor differentiation | 0.694 | 0.705 | 0.757 |  | 9.7 | 14.9 | 17.0 |
| Risk score (*n* = 213) | 0.706 | 0.724 | 0.776 |  | 10.8 | 16.7 | 18.6 |
| Risk score + Inflammation | 0.701 | 0.719 | 0.781 |  | 10.8 | 16.8 | 18.5 |

MVI microvascular invasion, AUC area under the curve.

**
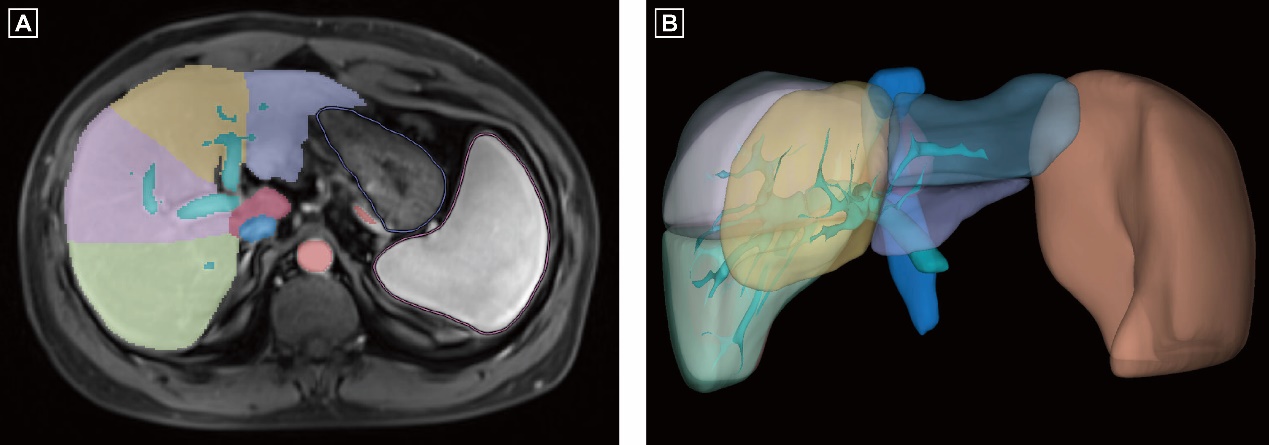
**

**Supplementary figure 1.** Fully-automated segmentation and calculation of liver and spleen volume using SenseCare software in a 42-year-old man. (**A**) Axial portal venous phase MR image shows segmentation of the liver (including each lobe and vessels) and spleen. (**B**) Three-dimensional reconstruction and calculation of a total liver volume of 1006.0 cm^3^ and a spleen volume of 638.1 cm^3^.

**
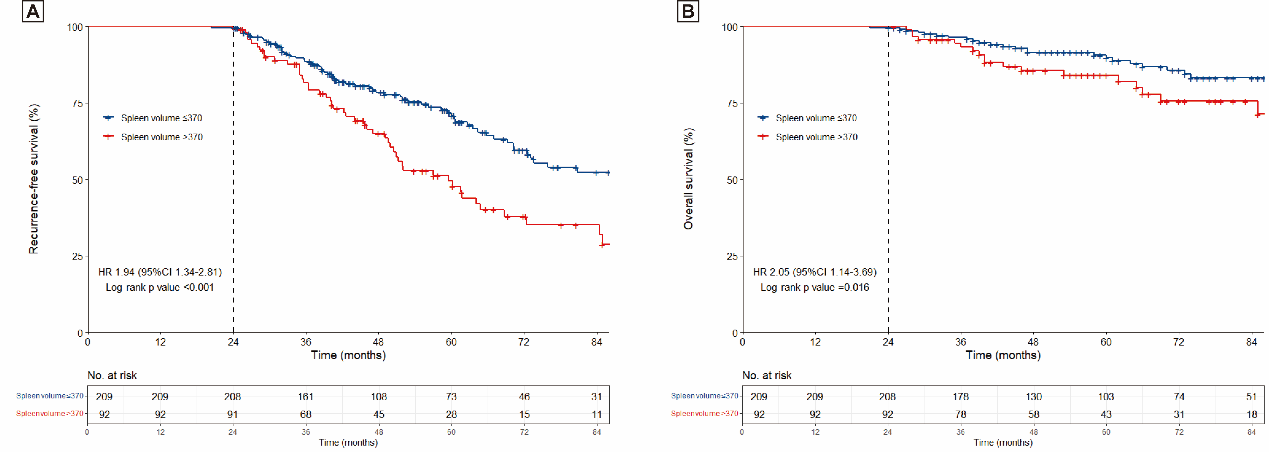
**

**Supplementary figure 2.** Kaplan-Meier curves of (**A**) recurrence-free survival, and (**B**) overall survival between two risk groups stratified according to baseline spleen volume (>370 vs. ≤370 cm^3^).

**
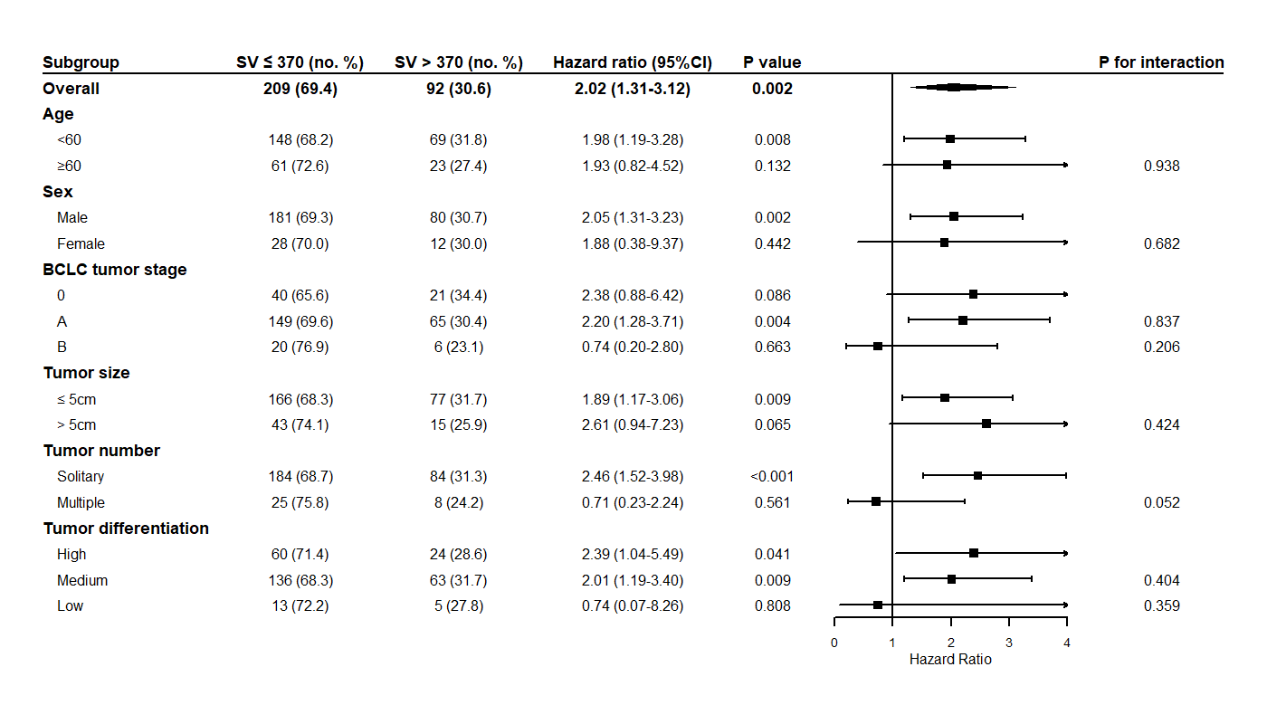
**

**Supplementary figure 3.** Forest plot demonstrating the effect of spleen volume on late recurrence in pre-specified subgroups.


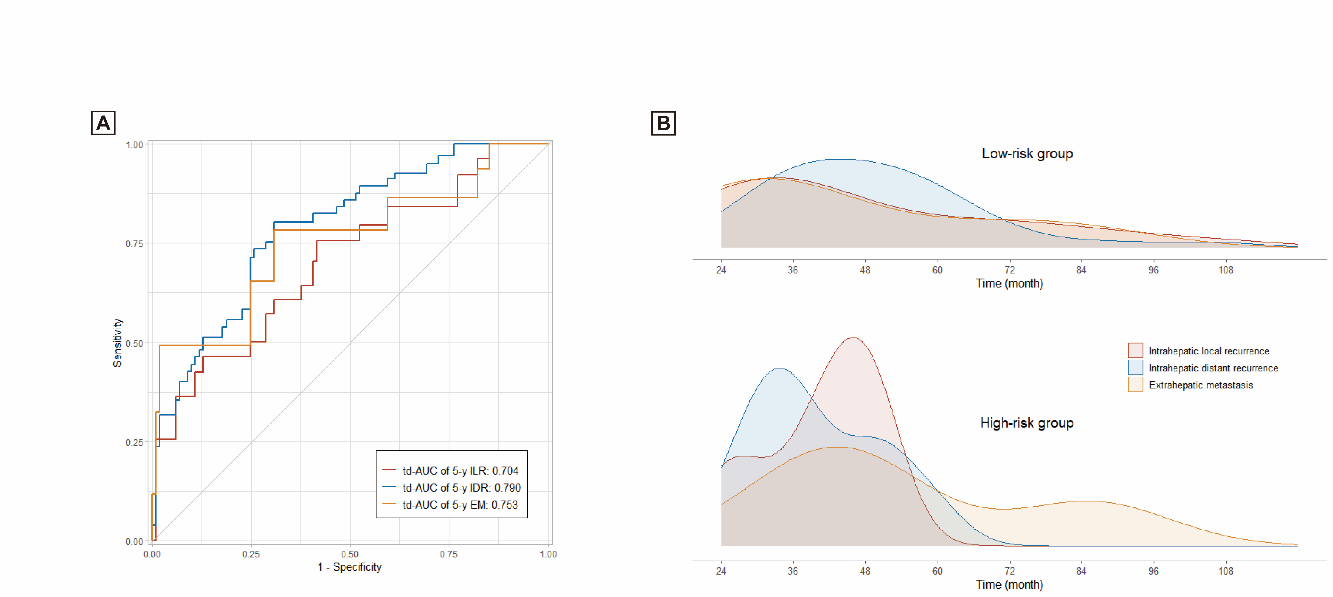


**Supplementary figure 4.** Prognostic value of the risk score in predicting different recurrence patterns. (A) Time-dependent area under the curve (td-AUC) of the risk score in predicting 5-year risk of intrahepatic local recurrence (ILR), intrahepatic distant recurrence (IDR), and extrahepatic metastasis (EM). (B) Density curves of three patterns of recurrence during follow-up between the low-risk and high-risk group.
